# Circulating Tumor DNA in Melanoma: Advances in Detection, Clinical Applications, and Integration with Emerging Technologies

**DOI:** 10.3390/ijms27031569

**Published:** 2026-02-05

**Authors:** Nicole Charbel, Joe Rizkallah, Mark Nicolas Bal, Amal El Masri, Elsa Armache, Malak Ghezzawi, Ali Awada, Lara Kreidieh, Jad Mehdi, Firas Kreidieh

**Affiliations:** 1 Division of Hematology and Oncology, Department of Internal Medicine, American University of Beirut, Beirut P.O. Box 11-0236, Lebanon; nc47@aub.edu.lb (N.C.); mnb25@mail.aub.edu (M.N.B.); axm09@mail.aub.edu (A.E.M.); eka05@mail.aub.edu (E.A.); ama221@mail.aub.edu (A.A.); lyk09@mail.aub.edu (L.K.); jmm44@mail.aub.edu (J.M.); 2 Department of Diagnostic Radiology, American University of Beirut, Beirut P.O. Box 11-0236, Lebanon; jr56@aub.edu.lb; 3 Department of Surgery, American University of Beirut, Beirut P.O. Box 11-0236, Lebanon; mg96@aub.edu.lb

**Keywords:** circulating tumor DNA, melanoma, minimal residual disease, recurrence, treatment monitoring, liquid biopsy

## Abstract

Circulating tumor DNA (ctDNA) has gained increasing attention as a non-invasive biomarker with potential utility across multiple stages of melanoma. ctDNA reflects tumor-derived genetic alterations in real time and has shown value in detecting minimal residual disease, identifying early recurrence, estimating tumor burden, and monitoring response to systemic therapies. In early-stage melanoma, postoperative ctDNA positivity is strongly associated with higher recurrence risk and often precedes radiologic detection. In advanced disease, ctDNA correlates with tumor volume and can distinguish responders from non-responders during targeted therapy and immunotherapy, while also identifying emerging resistance mechanisms. Despite these advantages, clinical implementation remains limited by low shedding in early-stage disease, variation among detection platforms, and the absence of standardized clinical thresholds. Recent advances, including fragmentomics, methylation assays, and multi-target sequencing strategies, aim to improve sensitivity, particularly in low-tumor-burden settings. Integration of ctDNA with radiomics, artificial intelligence, and digital pathology represents an additional opportunity to enhance precision in risk stratification and treatment adaptation. This review summarizes current evidence on ctDNA biology, detection methods, and clinical applications in melanoma and outlines ongoing challenges and future directions required for translation into routine practice.

## 1. Introduction

The incidence of melanoma has increased markedly over recent decades, with a threefold rise reported in the United States and similar trends observed globally [1]. Early detection has stabilized incidence among younger cohorts, but older adults continue to experience growing rates [2]. While localized melanoma carries an excellent prognosis, with a 5-year survival approaching 99.6%, advanced disease remains associated with high mortality [3]. Nevertheless, survival outcomes for Stage IV melanoma have improved substantially—from 15% in 2015 to 35.1%—reflecting the success of immune checkpoint inhibitors (ICIs) targeting PD-1 and CTLA-4, and targeted therapies against the BRAF and MEK pathways [4].

Melanoma development and progression are driven by recurrent alterations in key oncogenic signaling pathways. The mitogen-activated protein kinase (MAPK) pathway represents the dominant molecular driver, with activating mutations in *BRAF* (most commonly V600E/V600K) and *NRAS* promoting sustained signaling through MEK and ERK. Additional pathways implicated in melanoma biology include the phosphatidylinositol 3-kinase (PI3K)/AKT axis, which supports tumor survival, and frequent disruptions in cell-cycle regulation involving CDKN2A and CDK4. Immune-related mechanisms, including high tumor mutational burden and immune checkpoint signaling through PD-1/PD-L1 and CTLA-4, further influence disease behavior and therapeutic response. These molecular features underpin contemporary targeted and immune-based treatment strategies and provide a biological rationale for molecular monitoring approaches [5].

Current diagnostic approaches in melanoma rely primarily on clinical examination, dermoscopy, and histopathological assessment [6,7]. Dermoscopy enhances the visualization of pigmented lesions but relies heavily on operator expertise and may miss atypical or amelanotic variants. Histopathological assessment, while considered the gold standard, remains invasive and limited by sampling bias, providing only a static snapshot of a dynamic and spatially heterogeneous disease [8].

Similarly, serum biomarkers such as S100B, lactate dehydrogenase (LDH), and melanoma inhibitory activity (MIA) protein have been investigated for prognostic use, yet their low specificity and sensitivity—especially in early-stage disease or minimal residual disease (MRD)—restrict their clinical utility [9,10]. These shortcomings highlight the need for a non-invasive, dynamic biomarker capable of capturing disease evolution in real time.

Circulating tumor DNA (ctDNA) has emerged as a promising biomarker that fulfills these criteria. ctDNA consists of fragmented tumor-derived DNA released into the bloodstream, carrying clinically relevant mutations that inform diagnosis and guide therapy selection [11]. By capturing real-time genomic information, ctDNA enables early detection of recurrence, identification of resistance mechanisms, detecting progression and dynamic monitoring of treatment response—providing a minimally invasive complement to tissue-based testing. Accumulating evidence demonstrates that ctDNA can also detect MRD [11,12]. Several studies assessing ctDNA have indicated extended value beyond accurately detecting *BRAF* mutations, offering information about tumor heterogeneity and clonal evolution to guide personalized management [13,14].

Across multiple solid tumors, ctDNA has demonstrated clinical utility for molecular profiling when tissue is unavailable, detection of MRD, early identification of recurrence, and dynamic monitoring of treatment response. In colorectal, lung, and breast cancers, postoperative ctDNA positivity consistently predicts relapse risk and often precedes radiologic progression, while on-treatment ctDNA kinetics correlate with therapeutic efficacy and resistance emergence. These cross-cancer observations have accelerated the development of ctDNA-guided surveillance and adaptive treatment strategies, providing a strong rationale for similar applications in melanoma [15,16].

Melanoma represents a particularly compelling disease context for ctDNA-based biomarkers. It is characterized by a high tumor mutational burden, recurrent and well-defined driver mutations (e.g., *BRAF*, *NRAS*, *TERT* promoter), and pronounced intratumoral and intermetastatic heterogeneity. Tissue sampling is often limited by small primary lesions, inaccessible metastases, or repeated biopsies being clinically impractical, especially during systemic therapy. In addition, melanoma frequently exhibits atypical response patterns to immunotherapy, including pseudoprogression, where conventional imaging may be misleading. Together, these features make ctDNA uniquely suited for melanoma, where real-time, systemic molecular monitoring can complement imaging, capture clonal evolution, and improve treatment adaptation [4,11,17].

In this review, we synthesize current evidence on ctDNA in melanoma, focusing on three key aspects: (1) analytical and technological advances in ctDNA detection; (2) clinical applications of ctDNA across disease stages, including its role in MRD detection and treatment monitoring; and (3) future directions integrating ctDNA with radiomics, artificial intelligence, and other emerging tools to achieve adaptive, precision melanoma care.

## 2. Principles and Technologies of ctDNA Analysis

### 2.1. Biology and Origin of ctDNA

ctDNA represents a small fraction of the total cell-free DNA (cfDNA) present in plasma. It originates mainly from tumor-cell apoptosis and necrosis, which release fragmented double-stranded DNA molecules carrying somatic mutations identical to those in the primary tumor. Compared with cfDNA from normal cells, ctDNA fragments are typically shorter (<150 bp), whereas cfDNA shows a modal length of approximately 166 bp due to nucleosomal protection. The most frequently reported ctDNA targets in cutaneous melanoma include *BRAF* V600 and *NRAS* variants [18,19,20].

The proportion of ctDNA within the total pool of cfDNA—often referred to as the tumor fraction—is highly variable and reflects a complex interplay between tumor burden, vascularization, anatomical site of metastasis, and therapeutic status. Studies in melanoma have shown that ctDNA concentrations closely correlate with tumor burden and therapeutic response [20,21]. Metastatic lesions involving visceral organs, particularly the liver, release higher ctDNA levels than intracranial or subcutaneous sites, which may account for some false-negative results in ctDNA monitoring [22,23].

Moreover, dynamic changes in ctDNA closely mirror treatment response—rapid declines in ctDNA after therapy initiation are typically associated with favorable radiologic outcomes, whereas rising or re-emerging ctDNA levels often precede radiologic or clinical evidence of progression [20,21,24,25]. Because ctDNA aggregates molecular information from all tumor deposits, it provides a more comprehensive snapshot of tumor heterogeneity and clonal evolution than a single tissue biopsy [11,23].

A variety of technologies have been developed for ctDNA quantification in melanoma, differing in sensitivity, specificity, and genomic coverage. The principal platforms include digital droplet polymerase chain reaction (ddPCR), next-generation sequencing (NGS), and custom mutation panels.

### 2.2. Digital Droplet PCR

ddPCR remains a gold-standard method for the rapid and sensitive detection of known melanoma-specific mutations. By partitioning each sample into thousands of microdroplets and analyzing the fluorescent signal from each droplet, ddPCR allows absolute quantification of mutant and wild-type alleles without the need for standard curves. Using mutation-specific fluorescent probes, ddPCR reliably detects key melanoma driver alterations such as *BRAF* V600E/V600K and *NRAS* Q61. In a pivotal analysis, Sacco et al. detected the *BRAF* V600E mutation in 84.3% of plasma samples from patients with advanced melanoma, confirming the assay’s robustness in real-world settings [26]. Subsequent studies have shown that changes in ctDNA levels measured by ddPCR closely parallel clinical and radiologic responses, supporting its use for monitoring treatment efficacy and tumor progression [24,25]. Despite its strengths in accuracy and reproducibility, ddPCR’s major limitation lies in its restricted multiplexing capability, as only a small number of predefined loci can be assessed in a single run, limiting its application for broad mutational profiling or novel variant discovery [27,28].

### 2.3. Next-Generation Sequencing

NGS enables simultaneous analysis of multiple genes and the detection of low-frequency variants, thereby overcoming the primary limitation of ddPCR, which can only assess a few loci per assay [26]. Modern NGS platforms employing molecular barcoding, such as Tam-Seq, Safe-SeqS, and CAPP-Seq, enhance analytical sensitivity and reduce sequencing errors, allowing for detection of variants with mutant allele frequencies (MAF) below 1%, and providing sufficient cfDNA input (typically around 30 ng, or 4–5 mL of plasma) to ensure high-quality libraries. In melanoma, these improvements have yielded ctDNA detection rates approaching 84% in stage IV and 47% in stage III disease, with a lower detection threshold around 0.2% MAF [29].

Building on these advances, custom melanoma-specific sequencing panels have further expanded genomic coverage while maintaining high sensitivity. In one study, Diefenbach et al. designed a 123-amplicon panel covering 30 melanoma-associated genes, achieving high detection accuracy, and later refined this approach into a 15-gene panel encompassing key driver mutations, including the *TERT* promoter, with detection rates exceeding 60% [29,30]. These customized assays enable longitudinal monitoring of mutational evolution and resistance mechanisms across treatment courses.

Importantly, integrating NGS with ddPCR has demonstrated even greater diagnostic sensitivity and dynamic monitoring capacity, particularly in patients undergoing immunotherapy, by combining the quantitative precision of ddPCR with the broad genomic insight of NGS [27]. Together, these complementary methods offer a robust framework for comprehensive molecular surveillance in melanoma management. Understanding these biological and technical foundations is essential before contextualizing how ctDNA is applied across melanoma stages.

From a practical standpoint, cost remains an important consideration for clinical implementation. ddPCR has been reported to have 5–8.5-fold lower operational costs than NGS for ctDNA detection [31]. A formal micro-costing framework evaluating ctDNA workflows across platforms estimated per-sample costs ranging from approximately €168 to €7638 (USD $199–$9124), with ddPCR among the lowest-cost approaches and NGS-based panels among the most expensive, depending on assay complexity and testing volume [32]. In clinical implementation studies, NGS-based ctDNA assays are commonly priced in the range of approximately €600–€800 per test, with consumables representing the largest cost component [33]. As technologies mature and costs continue to decline, reimbursement policies, assay standardization, and cost-effectiveness analyses will remain key determinants of scalability in routine oncology practice.

### 2.4. ctDNA in Context: Comparison with Other Liquid Biopsy Biomarkers

Several liquid biopsy biomarkers have been explored in melanoma, including circulating tumor cells, exosomes, and circulating RNA species. Circulating tumor cells provide intact cellular material and enable phenotypic analyses; however, they are rare in melanoma, technically challenging to isolate, and show limited sensitivity in early-stage disease. Exosomes carry proteins, RNA, and DNA reflective of tumor biology, but their clinical application is constrained by complex isolation methods, lack of standardization, and limited assay reproducibility. In contrast, ctDNA offers higher analytical sensitivity, easier standardization, quantitative longitudinal tracking, and direct assessment of tumor-specific genomic alterations. These features have positioned ctDNA as the most clinically mature and scalable liquid biopsy modality in melanoma [6,23].

## 3. Clinical Applications of ctDNA in Melanoma

**Figure 1:**
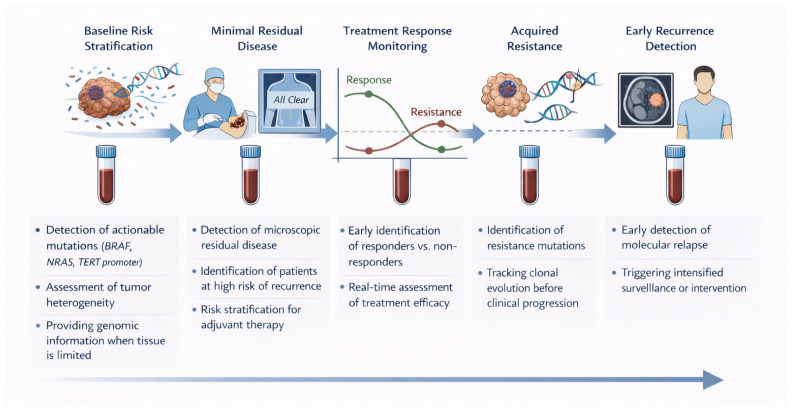
Clinical applications of circulating tumor DNA (ctDNA) across the cancer care continuum. ctDNA enables non-invasive genomic profiling at diagnosis, detection of minimal residual disease following curative-intent therapy, real-time monitoring of treatment response, identification of acquired resistance mutations, and early detection of molecular relapse prior to radiographic progression. Serial blood sampling supports longitudinal tracking of tumor dynamics, clonal evolution, and risk stratification, facilitating adaptive precision oncology strategies.

Beyond its analytical and technical foundations, ctDNA has emerged as a clinically relevant biomarker across multiple stages of melanoma. Its applications span risk stratification, treatment monitoring, detection of minimal residual disease, and early identification of recurrence, with distinct strengths and limitations depending on disease stage and clinical context. A summary of the principal clinical applications of ctDNA in melanoma is provided in Table 1 and Figure 1, which show that ctDNA enables longitudinal molecular monitoring across multiple stages of cancer management, including baseline genomic profiling, MRD detection, treatment response assessment, resistance tracking, and early recurrence detection.

Although cutaneous melanoma is typically accessible to excisional biopsy, tissue-based analysis provides only a static and spatially limited snapshot of tumor biology. ctDNA offers complementary diagnostic value by capturing real-time systemic tumor dynamics, intratumoral and intermetastatic heterogeneity, and clonal evolution during therapy—features not fully reflected in the primary lesion. This is particularly relevant in the adjuvant and surveillance settings, where ctDNA can identify molecular residual disease or early relapse despite complete surgical excision and negative imaging. Thus, rather than replacing histopathology, ctDNA enhances diagnostic and prognostic resolution across the disease course of cutaneous melanoma [11,15,17].

### 3.1. Early-Stage Melanoma

#### 3.1.1. Role of ctDNA in Staging, Treatment Monitoring and Detecting Disease Progression in Early-Stage Melanoma

ctDNA has emerged as a promising biomarker for staging, treatment monitoring, and early detection of disease progression in melanoma, including in early-stage settings. In early-stage melanoma, where traditional radiologic imaging may be limited in sensitivity, ctDNA offers a means of real-time tumor surveillance [11].

Although most validation studies focus on advanced melanoma, an expanding body of evidence now characterizes ctDNA dynamics in earlier-stage disease. For instance, ctDNA has been detected in a subset of stage IIB–IIC patients following surgery, and persistent or re-emerging ctDNA positivity has been shown to precede radiologic relapse by several weeks to months, indicating its role as an early marker of MRD [11,43]. Importantly, a 2024 meta-analysis evaluating postoperative ctDNA across resectable solid tumors, including stage I–III melanoma, reported a pooled hazard ratio of 7.48 for recurrence among ctDNA-positive patients, indicating a markedly increased risk of relapse compared with ctDNA-negative individuals. This finding highlights the strong prognostic value of MRD detection and suggests that ctDNA can identify patients at high risk of recurrence even when radiologic imaging shows no evidence of disease and overall tumor burden is low [15]. Collectively, these quantitative findings highlight ctDNA’s ability to detect biologically aggressive disease earlier than conventional surveillance.

Beyond staging, ctDNA also supports adjuvant treatment decisions. In early-stage melanoma—where escalation or de-escalation of therapy can be challenging—ctDNA offers a biologically grounded means to differentiate patients likely cured from those who may still harbor MRD despite negative imaging. This aligns with precision oncology principles, enabling treatment decisions to be informed by real-time molecular evidence rather than clinicopathologic factors alone [16].

Perioperative ctDNA dynamics further strengthen its relevance in early-stage disease. Preoperative ctDNA detection may be associated with greater tumor burden and biological aggressiveness, whereas postoperative or post-adjuvant ctDNA positivity correlates with MRD and relapse risk [26]. Studies of stage II and resected stage I–III melanoma cohorts have shown that the reappearance of ctDNA during surveillance is associated with a near-certain risk of future recurrence, frequently with a median lead time of approximately 3–5 months before radiologic detection [38,39].

Although randomized clinical trials are still ongoing to confirm the prognostic and predictive utility of ctDNA in early-stage melanoma, emerging qualitative data demonstrate high patient acceptability and enthusiasm for ctDNA-guided follow-up, with many patients viewing ctDNA as a reassuring, structured adjunct to standard surveillance strategies [44].

Collectively, current evidence indicates that ctDNA could meaningfully enhance risk stratification, guide adjuvant decision-making, and enable earlier detection of recurrence in early-stage melanoma. As assays become more sensitive and prospective trials mature, ctDNA is poised to become an integral component of routine management for patients with resectable melanoma.

#### 3.1.2. Challenges Due to Low Tumor Burden and Detection Sensitivity

The accurate detection of ctDNA in early-stage melanoma is hindered by both biological and technical factors, most of which arise from the intrinsically low tumor burden that characterizes early disease.

First, tumor biology and anatomical location influence the extent to which tumor DNA enters the bloodstream, often leading to very low plasma ctDNA concentrations that challenge assay sensitivity and make it challenging in early-stage disease [45]. Moreover, tumors situated in anatomically constrained compartments—such as the subcutaneous tissue or central nervous system—shed less ctDNA into systemic circulation, further reducing detection rates in early-stage disease [11].

In addition to these biological constraints, multiple technical limitations significantly affect the reliability of ctDNA assays in early-stage melanoma. A major challenge is the limited clinical validation of both the positive and negative predictive value of ctDNA testing in early-stage disease. While detectable ctDNA strongly suggests residual disease, a negative ctDNA result cannot be interpreted as evidence of cure, as residual disease may persist below the assay’s detection threshold. This limitation is compounded by the low number of tumor-derived DNA fragments in circulation; a single plasma sample may lack the necessary genome equivalents (GEs) to confidently rule out the presence of ctDNA. Achieving the tens of thousands of GEs needed for high-sensitivity detection is often impractical—or even impossible—in early-stage patients, given the limited quantity of cfDNA available [16].

Furthermore, current commercial ctDNA assays are generally less sensitive than tissue-based testing—not because of inherent analytical shortcomings, but due to the substantial blood volume, sequencing depth, and computational resources required to approximate tissue-level sensitivity via liquid biopsy [16]. These technical and logistical challenges collectively diminish the reliability of ctDNA testing in early-stage melanoma, especially when ctDNA is not detected.

Beyond issues of analytical sensitivity, lack of methodological standardization poses an additional barrier to clinical implementation. Variability in blood collection techniques, processing times, and storage conditions introduces inconsistency in sample quality, which directly affects assay performance and inter-study reproducibility. Compounding this, the absence of universally accepted thresholds for ctDNA positivity means that there is no consensus on what constitutes a clinically meaningful ctDNA level, limiting the biomarker’s utility for risk stratification or treatment decision-making in early-stage disease [46].

Physician hesitancy to adopt ctDNA testing in early-stage melanoma reflects these uncertainties. Survey data show that clinicians use ctDNA infrequently in this population, citing the low rate of detectable ctDNA after surgery and ambiguity regarding the interpretation of negative results as major deterrents [47].

Collectively, these biological, technical, and operational limitations highlight the need for more sensitive assays, standardized workflows, and rigorous clinical validation before ctDNA can be fully integrated into early-stage melanoma management.

#### 3.1.3. Strategies to Enhance ctDNA Detection Sensitivity

Given the limited detectability of ctDNA in early-stage melanoma, substantial efforts have focused on optimizing assay sensitivity through both technical innovations and methodological refinements. Enhancing sensitivity requires aligning assay design with the specific clinical context, as ctDNA tests are used for different purposes—including MRD detection, early response assessment, and monitoring of therapeutic efficacy. Accordingly, clinicians are encouraged to select or design assays based on disease stage, timing of sampling, and the clinical question being addressed [11].

Emerging technologies such as MRD-EDGE, which integrate fragmentomics with machine-learning–based signal enhancement, have shown particular relevance in early-stage disease, where ctDNA concentrations frequently fall below conventional detection thresholds [11]. Additional methodological advances, such as preferential selection of shorter DNA fragments, multi-target mutation tracking, refined computational pipelines, and methylation-based profiling, further strengthen assay sensitivity and robustness [11].

Fragment-size-based enrichment has demonstrated significant promise in low–tumor-burden settings. Isolating shorter fragments (90–150 bp) more than doubled ctDNA yield in over 95% of cases, enabling the detection of clinically relevant mutations and copy number alterations that standard workflows otherwise missed. When combined with shallow whole-genome sequencing, in vitro size selection markedly improved ctDNA detection and classification accuracy (AUC 0.97), supporting fragment-length profiling as a biologically informed, cost-effective enhancement strategy [48].

Furthermore, expanding the number and diversity of genomic or epigenomic targets, through broad multiplexed mutation panels or methylation signatures, substantially increases the likelihood of ctDNA detection in early-stage disease, where tumor shedding is minimal [16].

Collectively, these technological advancements underscore the potential of highly sensitive, multiplexed ctDNA assays—integrated with complementary biomarkers and supported by standardized pre-analytic workflows—to improve the accuracy and clinical utility of ctDNA-based detection in early melanoma. While early-stage melanoma presents unique challenges due to low ctDNA levels, the role of ctDNA becomes more established and clinically validated in advanced-stage disease.

### 3.2. Advanced-Stage Melanoma

ctDNA has demonstrated a pivotal role in the management of advanced-stage melanoma, functioning as a prognostic marker, a dynamic indicator of treatment response, and a sensitive tool for detecting disease progression. In a meta-analysis of more than 2000 patients with stage III–IV cutaneous melanoma, Gandini et al. reported that detectable baseline ctDNA was associated with significantly worse progression-free survival (PFS) (HR 2.47, 95% CI 1.85–3.29) and overall survival (OS) (HR 2.98, 95% CI 2.26–3.92), with comparable prognostic value in stage III and IV disease [34]. These data confirm the utility of ctDNA as a robust baseline risk stratifier.

In addition to its prognostic significance, ctDNA correlates strongly with tumor burden. Egger et al. demonstrated a positive association between plasma ctDNA levels and total tumor volume (R^2^ = 0.49, *p* < 0.001), with an even stronger correlation in patients experiencing radiologic progression (R^2^ = 0.91, *p* = 0.032) [21]. This supports ctDNA as a quantitative biomarker reflecting real-time disease load.

The dynamic monitoring of ctDNA has emerged as an effective measure of treatment response, often matching or surpassing traditional markers such as LDH or radiographic imaging. In one of the earliest studies, Gray et al. showed that baseline ctDNA predicted response to targeted therapies (vemurafenib, dabrafenib, or dabrafenib/trametinib) or immunotherapies (ipilimumab, nivolumab, pembrolizumab), and that ctDNA levels decreased significantly with effective MAPK inhibition (*p* < 0.001) [36]. Similarly, Braun et al. demonstrated that ctDNA kinetics within the first month of therapy accurately predicted treatment response, with detectable ctDNA at 30 days associated with inferior PFS in stage III patients (*p* = 0.018). The same study reported that ctDNA had a higher sensitivity for detecting active disease (76.8%) compared with LDH (53.6%) and S100 (46.4%), and that combining the three biomarkers increased sensitivity by an additional 9% [14].

Further evidence supports ctDNA as a powerful indicator of early molecular response. Among 142 metastatic melanoma patients treated with BRAF/MEK inhibitors or immunotherapies, declining ctDNA levels within the first 12 weeks strongly correlated with radiologic response (Cohen’s k = 0.798, *p* < 0.001) [35]. Marsavela et al. additionally found that ctDNA detection rates vary by treatment modality, with immunotherapy-associated ctDNA positivity substantially lower than with BRAF/MEK inhibitors (*p* = 0.0149), reflecting different patterns of tumor cell death and molecular shedding [35].

ctDNA has also proven valuable for identifying resistant clonal subtypes and early treatment failure [36,49,50]. Herbreteau et al. showed that increases in ctDNA within 2–4 weeks of anti-PD-1 therapy initiation were associated with markedly poor outcomes (4-month PFS 0%, 1-year OS 13%), underscoring the ability of ctDNA kinetics to detect early resistance [49]. A 2021 meta-analysis by Zheng et al. (16 studies, 1781 patients) further highlighted that high baseline ctDNA predicted inferior treatment response (pooled HR = 3.29, 95% CI 1.73–6.25) [49]. Di Nardo et al. confirmed this association, showing that each unit increase in ctDNA from baseline to follow-up increased the risk of progression by 24% (OR = 1.24, *p* = 0.020), whereas patients with declining ctDNA had significantly better OS and PFS (*p* < 0.001) [51].

Serial ctDNA monitoring also correlates strongly with radiologic assessments across modalities including CT, MRI, and PET/CT, as defined by RECIST 1.1 and iRECIST criteria [17,40]. Marsavela et al. reported higher ctDNA detection rates in patients with visceral metastases at progression, while lower rates were observed in those with lung, lymph node, or intracranial disease, reflecting site-specific differences in ctDNA shedding. Importantly, ctDNA may help distinguish true progression from pseudoprogression in immunotherapy-treated patients. Lee et al. demonstrated that all patients with pseudoprogression exhibited low or favorable ctDNA profiles, whereas most with true progression had rising or high ctDNA levels [17]. This distinction is clinically critical to avoid premature therapy discontinuation.

Ongoing work aims to standardize ctDNA interpretation and integrate it into clinical decision-making. Jakobsen et al. recently proposed the “ctDNA-RECIST” classification, a molecular response framework that correlates strongly with survival outcomes and may complement or refine traditional radiologic criteria [42].

Collectively, these findings underscore ctDNA as a powerful, dynamic biomarker in advanced melanoma, capable of predicting prognosis, monitoring treatment response, identifying molecular resistance, and distinguishing true from false progression.

#### ctDNA Applications in Uveal Melanoma

Uveal melanoma represents a biologically and clinically distinct entity from cutaneous melanoma, arising from melanocytes of the uveal tract and characterized by unique driver alterations, most commonly involving *GNAQ*, *GNA11*, *BAP1*, *SF3B1*, and *EIF1AX*. Unlike cutaneous melanoma, uveal melanoma is not routinely accessible to excisional biopsy, as primary ocular tumors are often treated with eye-preserving radiotherapy without tissue removal. Consequently, molecular characterization frequently relies on fine-needle aspiration or is not performed at all. In this context, ctDNA liquid biopsy provides a particularly valuable alternative for molecular profiling, prognostication, and longitudinal disease monitoring. Multiple studies have demonstrated that ctDNA levels in uveal melanoma correlate strongly with metastatic burden and overall survival, particularly in metastatic disease, and that early ctDNA dynamics may outperform radiologic criteria in predicting treatment benefit, including during tebentafusp therapy. These features position ctDNA as a critical tool in uveal melanoma, where conventional tissue-based genomics is limited and early detection of systemic relapse remains a major unmet need [23,41,52].

### 3.3. Recurrence Monitoring

Building on the principles described in earlier sections, ctDNA’s most established clinical application is in recurrence monitoring. Its ability to provide a real-time, minimally invasive evaluation of tumor burden offers a significant advantage over traditional surveillance methods, such as imaging, which have limitations in detecting microscopic disease and carry risks associated with radiation exposure and cost. The detection of ctDNA in the bloodstream of patients who have undergone definitive treatment for melanoma is a strong indicator of MRD and a powerful predictor of subsequent clinical recurrence. This section will examine the dynamics of ctDNA during and after treatment, the technological evolution of its detection, the biological factors governing its presence, and its role in identifying MRD and predicting relapse.

#### 3.3.1. Longitudinal ctDNA Dynamics During and Post-Treatment

The monitoring of ctDNA levels over time, known as longitudinal analysis, provides a dynamic view of treatment response and disease status. The very short half-life of ctDNA in the circulation, estimated to be less than two hours, means that its levels provide a near-real-time representation of the viable tumor burden. In patients with resected high-risk melanoma, the presence of detectable ctDNA before the initiation of adjuvant therapy is a significant prognostic marker for early recurrence [37]. A landmark 2025 biomarker analysis from the COMBI-AD trial demonstrated that in patients with resected Stage III *BRAF* V600-mutant melanoma, baseline ctDNA detection was associated with a substantially lower recurrence-free survival rate [37]. This study highlights the utility of baseline ctDNA levels in risk stratifying patients for adjuvant therapy.

The dynamics of ctDNA during treatment are equally informative. A rapid decline in ctDNA levels or its complete clearance is often indicative of a favorable response to therapy, including immunotherapy and targeted treatments. This is supported by a multicenter retrospective study which found that ctDNA clearance after 6–8 weeks of anti-PD-1 based therapy was associated with significantly improved survival [53], a finding now reinforced on a larger scale by a meta-analysis focused on patients with melanoma receiving ICIs. This analysis confirmed that pre-treatment ctDNA positivity was strongly associated with poorer overall survival (hazard ratio [HR] = 3.19) and progression-free survival (HR = 2.08), whereas on-treatment clearance predicted better outcomes. Similarly, ctDNA positivity during treatment was also associated with poorer overall survival (HR = 4.57) and progression-free survival (HR = 3.79) [54]. Conversely, a stable or rising ctDNA level during therapy, or the new appearance of detectable ctDNA after initial clearance, can signal primary or acquired treatment resistance and disease progression. This often precedes radiological evidence of relapse by weeks to months [37], a phenomenon termed molecular relapse, which presents a critical window of opportunity for clinical intervention, such as switching therapeutic agents before the macroscopic disease burden increases.

Post-treatment surveillance is another area where longitudinal ctDNA monitoring shows considerable promise. In patients who have completed therapy and are clinically disease-free, the reappearance of ctDNA can be an early indicator of recurrence. A 2025 study highlighted that nearly all patients with detectable ctDNA at follow-up appointments experienced melanoma recurrence [37]. This early detection can prompt physicians to escalate surveillance with more frequent imaging or even consider pre-emptive therapeutic strategies, fundamentally shifting the approach from reactive to proactive management of melanoma recurrence [38].

#### 3.3.2. Identification of MRD and Recurrence Prediction

MRD refers to the presence of a small number of cancer cells in the body after treatment that are undetectable by conventional imaging techniques. ctDNA serves as a highly specific biomarker for MRD, as it is composed of tumor-derived DNA fragments [15]. Numerous studies have now established that longitudinal ctDNA monitoring is a powerful method for detecting MRD in surgically resected melanoma [39].

A critical evolution in MRD detection has been the shift from earlier detection methods, like ddPCR, to highly sensitive, tumor-informed bespoke assays using NGS. Although ddPCR is highly sensitive for known, single hotspot mutations (e.g., *BRAF* V600E), its utility is limited to patients harboring that specific mutation and it is susceptible to being missed if the clonal evolution of the tumor relies on other driver mutations. In contrast, tumor-informed bespoke panels are created by first sequencing the primary tumor to identify a unique signature of 16 to 50 clonal somatic mutations. The assay is then personalized to track these specific mutations in the plasma. This multi-target approach dramatically increases the analytical sensitivity and specificity, enabling the detection of low variant allele frequencies [55].

This technological superiority is validated by recent studies. A key 2025 study by Genta et al. utilizing the bespoke RaDaR assay found that post-surgical MRD detection was associated with worse overall survival and importantly, that 26% of ctDNA-positive patients were *BRAF*/*NRAS* wild-type, a population that would be entirely missed by common hotspot ddPCR assays [56]. This study also confirmed a significant lead time, with MRD detection preceding radiological relapse by a median of 4 months [56]. Further validating this approach, an analysis from the KEYNOTE-942 trial showed that baseline ctDNA positivity, as detected by a bespoke assay, was associated with a dramatically shorter recurrence-free survival (HR for recurrence in ctDNA^+^ vs. ctDNA^−^ patients = 0.150) [57]. These advanced assays strengthen the conclusions of broader meta-analyses, such as a review of 80 studies that found postoperative ctDNA positivity carried an HR for recurrence of 7.48 [15].

However, the detection of ctDNA is not absolute and is influenced by complex biological factors. The rate of ctDNA shedding into the bloodstream varies significantly based on tumor biology and location. Factors influencing shedding include tumor size, proliferation rate, cellular turnover (apoptosis and necrosis), and vascularization. Furthermore, the anatomical site of metastasis is a major determinant of ctDNA detectability. For instance, metastases in highly vascularized organs like the liver and lungs tend to shed more ctDNA than disease confined to the skin or muscle. The central nervous system represents a particular challenge. The blood–brain barrier can sequester ctDNA, leading to significantly lower or even undetectable levels in peripheral blood, even in the presence of substantial intracranial disease. This was highlighted in a 2025 study that reported lower sensitivity for brain metastases (33.3%) compared to liver or lymph node metastases (87.5%) [58]. This highlights the need for caution when interpreting a negative ctDNA result in patients at high risk for brain metastases and may necessitate parallel analysis of cerebrospinal fluid.

The prognostic power of ctDNA-based MRD detection is now being positioned as a critical tool for patient stratification and tailoring adjuvant therapy decisions [56,59]. The ultimate goal, however, is to use this information to improve patient outcomes, a question that requires evidence from prospective, interventional trials. The next wave of clinical studies is designed to answer this question directly. For instance, the DETECTION trial (NCT04901988) is randomizing ctDNA-positive patients after surgery to either standard surveillance or early intervention with immunotherapy, which will determine if acting on MRD detection improves survival [60].

The integration of these assays into routine clinical care is not without challenges. The cost-effectiveness of serial monitoring, particularly with expensive bespoke assays, needs formal evaluation. Standardization of blood collection, processing, and analytical protocols is essential for reproducibility across different centers. Finally, the clinical and ethical dilemma of how to treat a molecular relapse in an otherwise asymptomatic, radiologically negative patient remains. The results of ongoing interventional studies will be pivotal in resolving these issues and formally integrating ctDNA-guided strategies into the standard of care for melanoma management. While ctDNA offers unparalleled sensitivity for recurrence detection, integrating it with emerging data modalities may further enhance predictive accuracy.

Selected prospective studies and clinical trials evaluating ctDNA in melanoma across disease stages are summarized in Table 2. To contextualize ctDNA within the broader biomarker landscape across melanoma disease stages, key biomarkers and their clinical utility are summarized in Table 3.

## 4. Emerging Approaches and Future Directions

### 4.1. Integration of Artificial Intelligence

Artificial intelligence (AI), particularly through machine learning (ML) and deep learning algorithms, has shown promise in processing the vast datasets derived from ctDNA analyses in melanoma. AI models can integrate large-scale biological datasets, including ctDNA mutational profiles, radiomics, and cytokine levels, in order to support clinical decision-making and personalize treatment plans.

Goussault et al. developed and validated four ML models using demographic, clinical, and mutational data from over 900 melanoma patients to predict response to immunotherapy and targeted therapies. These models, trained on real-world data, effectively stratified patients into responders and non-responders, demonstrating translational potential [62,63]. Radiomics-based ML approaches are also increasingly applied to non-invasive mutational prediction. For example, ML analysis of MRI radiomic features has achieved high accuracy in predicting *BRAF* mutation status in brain metastases, potentially limiting the need for invasive biopsies [64]. Similarly, Tabari et al. combined radiomic imaging data with LDH levels to enhance ML model performance for predicting ICIs response, underscoring the value of multi-modal data integration [65]. Beyond imaging and ctDNA, AI has been applied to digital histopathology. Deep convolutional neural networks (DCNNs) have been trained on routine H&E slides to predict survival and immunotherapy outcomes in melanoma patients. These models autonomously assess tumor regions without requiring cell-specific annotations or immunohistochemical staining, thus offering scalable and cost-efficient predictive capabilities [66]. Unlike earlier models reliant on CD3^+^/CD8^+^ cell labeling, newer approaches evaluate whole-slide images (WSIs) directly and retain predictive performance across tissue types, staining protocols, and scanning platforms [67].

Despite the transformative role of immune checkpoint blockade in melanoma treatment, a significant proportion of patients fail to achieve durable benefit. Traditional predictors—such as PD-L1 expression or tumor mutation burden (TMB)—have important limitations. PD-L1 expression is dynamic and inducible, reducing its predictive utility post-treatment initiation, while the prognostic relevance of TMB remains debatable, especially in melanoma where results across studies have been inconsistent [67]. Recent advancements in AI-based predictors aim to overcome these limitations. For instance, Chen et al. demonstrated that exosomal PD-L1 changes could predict response with high accuracy, though widespread use is constrained by technical barriers in exosome isolation [68].

In contrast, AI-powered histologic classifiers offer several advantages. Harder et al. and other groups have shown that DCNNs trained on digital pathology slides can reliably stratify patients based on predicted treatment outcomes, with minimal dependence on biopsy site or slide age [69]. Their models achieved sensitivities and specificities comparable to, or even exceeding, PD-L1 IHC assays used in several clinical trials. Moreover, their performance was consistent across therapies targeting CTLA-4 and PD-1, suggesting that the underlying AI-detected biological features may surpass specific checkpoint pathways. This may be due to the networks’ capacity to detect features such as increased nuclear size and density—thus reflecting genome instability and neoantigen load, both of which influence immunotherapy responsiveness [69]. Brohet et al. further support this by highlighting the promise of ML classifiers trained on real-world treatment response data. Their pilot study demonstrated that survival and response in advanced melanoma could be reasonably predicted using clinical and cytokine-based input features, though the authors caution that small sample sizes and limited biomarker inclusion necessitate further prospective validation [62]. In addition to histologic and cytokine-based models, Wang et al. applied ML to cytokine clearance patterns during treatment, associating low-clearance signatures with favorable survival outcomes. Although their applicability in routine clinical settings has yet to be fully established, these findings underline AI’s strength in recognizing dynamic treatment-related immune trends—an area where static biomarkers often fall short [70].

### 4.2. Combining ctDNA with Imaging

ctDNA dynamics have emerged as a powerful biomarker for monitoring treatment response across melanoma subtypes. In metastatic uveal melanoma (mUM) treated with tebentafusp, an early ctDNA decline (≥0.5-log reduction) was associated with markedly longer overall survival (16.9 vs. 8.5 months), even in patients classified as having progressive disease under RECIST criteria, underscoring the limitations of size-based radiologic assessment alone [52].

Radiomics provides an important complementary tool. Standard CT and MRI scans contain extensive quantitative information regarding tumor burden, heterogeneity, and morphology that is not captured by conventional dimension-based measurements [41]. Radiomic signatures derived from baseline and early follow-up imaging have been shown to predict long-term benefit from tebentafusp in mUM, aligning closely with ctDNA dynamics. Features such as tumor homogeneity and entropy on week-eight imaging correlated strongly with ctDNA reduction, achieving a predictive accuracy of AUC 0.70 [41].

Beyond mUM, radiomic features reflecting lesion heterogeneity and compact morphological patterns have been predictive of immunotherapy response in metastatic melanoma and non-small cell lung cancer patients treated with PD-1 inhibitors, independent of anatomical site [61]. Radiomics also has the capacity to capture immune-relevant features: a radiomic signature of tumor-infiltrating CD8 cells—validated against transcriptomic data—successfully predicted immune phenotype and clinical outcomes in patients receiving PD-1/PD-L1 blockade [71].

Together, these findings highlight the complementary value of each modality: ctDNA serves as a systemic marker of tumor burden and biological activity, whereas radiomics provides spatially resolved insight into tumor architecture and microenvironmental features. Integrating both approaches offers a noninvasive, multidimensional biomarker platform for immunotherapy monitoring.

This combined strategy may refine treatment stratification and clinical decision-making. First, it enables earlier recognition of responders; in mUM, the combination of week-eight radiomic features with ctDNA decline predicted long-term benefit from tebentafusp, overcoming the inability of RECIST to capture atypical immunotherapy response kinetics [41]. Second, it provides a multilayer characterization of tumor biology: ctDNA reflects systemic dynamics, while radiomics captures intratumoral heterogeneity, morphology, and immune-cell infiltration—key determinants of immunotherapy efficacy [71,72]. Third, ctDNA–radiomics integration supports personalized management strategies. Patients who exhibit radiographic progression but sustained ctDNA reduction and favorable radiomic signatures may safely continue therapy, whereas those lacking both signals may benefit from early therapeutic modification [41].

Finally, radiomics features associated with CD8^+^ T-cell infiltration suggest that integrating ctDNA with imaging could function not only as a prognostic biomarker but also as a surrogate for tumor–immune interactions, guiding rational treatment sequencing or combinatorial approaches [71,73]. This multidimensional and noninvasive framework holds significant promise for advancing precision oncology in melanoma.

### 4.3. Emerging Technologies and Biomarker Discovery Advancements

Recent years have witnessed substantial progress in non-invasive technologies aimed at improving melanoma detection, risk stratification, and ctDNA-based biomarker discovery. Advances in digital imaging—including total body photography, 3D imaging, and serial dermoscopy—now enable more precise longitudinal tracking of neoplastic changes in high-risk individuals. When augmented by artificial intelligence (AI), these tools can enhance diagnostic accuracy, enable earlier detection, and reduce unnecessary biopsies [74]. Convolutional neural networks (CNNs) trained on large dermoscopic image datasets have achieved diagnostic performance comparable to, and in some cases surpassing, expert dermatologists, particularly when combined with clinical metadata. Increasingly, these CNN-based platforms are being integrated with ctDNA analyses to flag high-risk lesions that may harbor actionable genomic alterations [66].

Emerging molecular diagnostic techniques—including adhesive patch testing, photoacoustic imaging, and Raman spectroscopy—further complement ctDNA applications by offering real-time, spatially resolved assessment of tumor biology. When coupled with AI-driven analytical frameworks, these modalities open new opportunities for early melanoma detection, refined risk stratification, and continuous disease monitoring [74].

Digital histopathology has also become a particularly impactful domain. DCNNs applied to WSIs have demonstrated robust predictive power across diverse slide scanners and staining protocols, enabling scalable AI-based diagnostic support, including in resource-limited settings. By reducing reliance on repeat biopsies or complex molecular assays, these approaches may shorten diagnostic timelines and facilitate more timely therapeutic interventions [67].

ctDNA analysis can also be integrated with other liquid biopsy modalities to create a more comprehensive molecular and cellular diagnostic framework. Circulating tumor cells provide intact cellular material that enables phenotypic, transcriptomic, and functional analyses, while tumor-derived extracellular vesicles and exosomes carry proteins, RNA, and DNA reflective of tumor–microenvironment interactions. Although each modality has individual limitations, combined approaches have demonstrated complementary value, with ctDNA offering high analytical sensitivity for genomic alterations and circulating tumor cells or vesicles providing spatial and functional context. Integrated liquid biopsy strategies may therefore enhance disease characterization, improve resistance detection, and refine prognostic stratification in melanoma, particularly in advanced or treatment-resistant settings [6,13].

Collectively, the convergence of AI, digital pathology, ctDNA technologies, and novel molecular biomarkers is poised to transform personalized melanoma care. With continued validation and integration into real-world clinical workflows, these innovations may soon form the backbone of AI-guided oncology across both academic and community practice environments.

### 4.4. Prospects for Personalized and Adaptive ctDNA-Guided Treatments

As ctDNA technologies continue to mature, one of the most promising future directions in melanoma care is the development of personalized and adaptive treatment strategies driven by real-time molecular monitoring. However, to fully integrate ctDNA into routine care, several key areas require advancement.

First, standardization of ctDNA assays remains essential. This includes defining analytical sensitivity, establishing clinically meaningful thresholds, and harmonizing laboratory workflows across institutions—steps highlighted as necessary for advancing precision oncology in ctDNA-based treatment paradigms [75].

Second, prospective trials are needed to test adaptive therapy algorithms driven by ctDNA trends. Potential applications include escalating therapy when ctDNA remains detectable post-treatment, pausing or de-intensifying therapy when ctDNA becomes persistently undetectable, and switching systemic regimens in response to ctDNA-identified resistance mutations. These strategies hold the potential to personalize dose intensity, minimize toxicity, and optimize long-term outcomes, but require rigorous clinical validation [76].

Third, expanding ctDNA into early-stage and non-metastatic melanoma is an important frontier. Emerging evidence shows that ctDNA can detect MRD and predict recurrence in non-metastatic melanoma, supporting its future use in guiding adjuvant therapy decisions and early intervention strategies [77].

Fourth, ctDNA will likely be integrated with complementary biomarkers—including immune profiling, circulating proteins, or TMB—as well as imaging-based tools and digital pathology platforms. Such multi-modal frameworks are expected to improve risk stratification and enable more precise detection of early therapeutic resistance [75].

Finally, widespread adoption will depend on addressing broader implementation challenges, including clinical utility, cost-effectiveness, and workflow integration. As outlined by Ignatiadis et al., the translation of liquid biopsy into everyday oncology practice will require coordinated efforts in validation, regulatory approval, and clinical guideline development [78].

Collectively, these future directions position ctDNA to evolve from a research biomarker into a central component of adaptive, personalized melanoma care, enabling earlier intervention, reduced overtreatment, and more refined therapeutic decision-making.

## 5. Conclusions

ctDNA has emerged as a transformative biomarker in melanoma management, offering a minimally invasive means to monitor disease burden, detect MRD, assess treatment response, and identify emerging resistance. Advances in ddPCR, next-generation sequencing, and tumor-informed bespoke assays have expanded its clinical utility across both early and advanced disease stages. When integrated with radiomics, artificial intelligence, and digital pathology, ctDNA enables a multidimensional assessment of tumor behavior that enhances risk stratification and supports more personalized treatment decisions.

Among available biomarkers, ctDNA currently represents the most clinically mature and promising liquid biopsy approach in melanoma, particularly for MRD-guided adjuvant therapy, early detection of recurrence, and real-time monitoring of immunotherapy response. The strongest near-term clinical applications include identifying patients at high relapse risk after surgery, distinguishing true progression from pseudoprogression, and adapting therapy based on dynamic ctDNA kinetics.

Despite its promise, key challenges remain, including assay standardization, sensitivity in low-tumor-burden settings, cost-effectiveness, and the need for prospective interventional validation. Continued integration of ctDNA into multidisciplinary clinical workflows, alongside imaging- and immune-based biomarkers, will be essential for translating its potential into routine practice. As evidence matures and implementation barriers are addressed, ctDNA is poised to evolve from a complementary biomarker into a central pillar of personalized melanoma care.

## Figures and Tables

**Table 1:** Summary of clinical applications and use cases of ctDNA in melanoma.

Clinical Context	Application of ctDNA	Clinical Utility	Key Limitations	Representative References
Initial diagnosis and molecular profiling	Detection of driver mutations (e.g., *BRAF*, *NRAS*, *TERT* promoter)	Non-invasive identification of actionable mutations when tissue is unavailable or insufficient; assessment of tumor heterogeneity	Limited sensitivity in low-tumor-burden disease; cannot replace histopathology	[11,26]
Baseline risk stratification (advanced disease)	Quantification of baseline ctDNA levels	Prognostic stratification for progression-free and overall survival; correlation with tumor burden	Variability in shedding by metastatic site; lack of standardized cut-offs	[21,34]
Treatment response monitoring	Serial ctDNA measurements during systemic therapy	Early identification of responders and non-responders; real-time assessment of therapeutic efficacy	Transient fluctuations may occur; interpretation requires longitudinal trends	[14,35]
Detection of acquired resistance	Identification of emerging resistance mutations	Early molecular detection of treatment resistance before radiologic progression	Requires broad sequencing panels; higher cost and technical complexity	[27,36]
Minimal residual disease after surgery	Postoperative ctDNA detection	Identification of residual microscopic disease; strong prediction of recurrence risk	False negatives in early-stage disease; limited sensitivity with low ctDNA levels	[15,37]
Recurrence surveillance	Longitudinal ctDNA monitoring after definitive therapy	Earlier detection of relapse compared with imaging; enables pre-emptive clinical intervention	Optimal surveillance intervals not yet standardized	[38,39]
Distinguishing true progression from pseudoprogression	ctDNA kinetics during immunotherapy	Supports differentiation between immune-related pseudoprogression and true disease progression	Not reliable in isolated intracranial disease	[17,40]
Integration with imaging and AI	Combined ctDNA, radiomics, and ML models	Improved risk stratification, response prediction, and adaptive treatment decisions	Requires validation, interoperability, and clinical workflow integration	[41,42]

AI, artificial intelligence; ctDNA, circulating tumor DNA; ML, machine learning.

**Table 2:** Selected clinical trials and prospective studies evaluating ctDNA in melanoma.

Study/Trial	Disease Stage	ctDNA Methodology	Number of Patients	Clinical Application	Key Findings	Reference
COMBI-AD biomarker analysis	Resected stage III (*BRAF* V600)	ddPCR (*BRAF* V600)	870	Prognosis; MRD	Baseline ctDNA positivity associated with significantly worse recurrence-free survival and overall survival	[37]
KEYNOTE-942 (ctDNA analysis)	Resected high-risk melanoma	Tumor-informed NGS (bespoke)	157	MRD; recurrence prediction	Baseline ctDNA positivity strongly associated with early recurrence; ctDNA-negative patients showed prolonged recurrence-free survival	[57]
Longitudinal MRD monitoring study	Stage I–III melanoma after surgery	Tumor-informed NGS	66	MRD detection	ctDNA re-emergence preceded radiologic recurrence by a median of ~ 3–5 months	[38]
Longitudinal ctDNA surveillance	Resected stage II–III melanoma	Tumor-informed NGS	69	MRD; surveillance	Postoperative ctDNA positivity predicted recurrence with high specificity	[39]
Anti–PD-1 monitoring (multicenter retrospective)	Stage III–IV	ddPCR and NGS	142	Treatment response	Early ctDNA clearance associated with improved progression-free and overall survival	[51]
Anti–PD-1 early kinetics study	Metastatic melanoma	ddPCR	49	Early resistance detection	Rising ctDNA within 2–4 weeks predicted poor response and survival	[49]
Tebentafusp phase II trial (uveal melanoma)	Metastatic uveal melanoma	ddPCR/NGS	127	Response monitoring	Early ctDNA decline correlated with overall survival, outperforming RECIST	[52]
DETECTION trial (ongoing)	Stage IIB–IIC post-surgery	Tumor-informed ctDNA	Planned ~500	ctDNA-guided therapy	Randomized trial testing early intervention based on ctDNA positivity	[60]

ctDNA, circulating tumor DNA; ddPCR, droplet digital polymerase chain reaction; MRD, molecular residual disease; NGS, next-generation sequencing.

**Table 3:** Biomarkers evaluated across disease stages of melanoma and their clinical utility.

Disease Stage	Biomarker	Sample Type	Clinical Application	Strength of Evidence	Key Limitations	Representative References
Early-stage melanoma	ctDNA (tumor-informed NGS)	Plasma	Detection of minimal residual disease; recurrence risk stratification	High (prospective studies and meta-analyses)	Limited sensitivity at very low tumor burden; false negatives possible	[15,39]
Early-stage melanoma	S100B	Serum	Prognostic marker during follow-up	Moderate	Low sensitivity and specificity; limited MRD utility	[9]
Early-stage melanoma	LDH	Serum	Baseline prognostic marker	Low	Poor sensitivity in early disease; non-specific	[10]
Advanced-stage melanoma	ctDNA kinetics	Plasma	Treatment response monitoring; early resistance detection	High	Variability by metastatic site; requires serial sampling	[14,35]
Advanced-stage melanoma	LDH	Serum	Prognosis; disease burden estimation	Moderate	Limited dynamic sensitivity; late marker	[34]
Advanced-stage melanoma	Radiomics	Imaging-derived	Response prediction; outcome stratification	Emerging	Requires validation and standardization	[61]
Recurrent melanoma	ctDNA re-emergence	Plasma	Early detection of molecular relapse	High	Reduced sensitivity in isolated intracranial disease	[17,38]
Recurrent melanoma	ctDNA clearance	Plasma	Assessment of treatment efficacy	High	Interpretation requires longitudinal trends	[51]
Recurrent melanoma	Circulating tumor cells	Whole blood	Cellular characterization of relapse	Low-moderate	Low detection rate; technical complexity	[6]

ctDNA, circulating tumor DNA; LDH, lactate dehydrogenase; MRD, molecular residual disease; NGS, next-generation sequencing.

## Data Availability

No new data were created or analyzed in this study. Data sharing is not applicable to this article.

## Abbreviations

The following abbreviations are used in this manuscript:

AI	Artificial intelligence
bp	Base pairs
CI	Confidence interval
cfDNA	Cell-free DNA
CNN	Convolutional neural network
ctDNA	Circulating tumor DNA
DCNN	Deep convolutional neural network
ddPCR	Digital droplet polymerase chain reaction
GE	Genome equivalent
H&E	Hematoxylin and eosin
HR	Hazard ratio
ICI	Immune checkpoint inhibitor
LDH	Lactate dehydrogenase
MAF	Mutant allele frequency
ML	Machine learning
MRD	Minimal residual disease
mUM	Metastatic uveal melanoma
NGS	Next-generation sequencing
OS	Overall survival
PFS	Progression-free survival
TMB	Tumor mutation burden
